# *Galleria mellonella* for the Evaluation of Antifungal Efficacy against Medically Important Fungi, a Narrative Review

**DOI:** 10.3390/microorganisms8030390

**Published:** 2020-03-11

**Authors:** Sana Jemel, Jacques Guillot, Kalthoum Kallel, Françoise Botterel, Eric Dannaoui

**Affiliations:** 1 EA Dynamyc UPEC, EnvA, USC Anses, Faculté de Médecine de Créteil, 94000 Créteil, France; jemelsana.benayed@gmail.com (S.J.); jguillot@vet-alfort.fr (J.G.); francoise.botterel@aphp.fr (F.B.); 2 Université Tunis EL Manar, Faculté de médecine de Tunis, Tunis 1007, Tunisie; kallelkalthoum@gmail.com; 3 UR17SP03, centre hospitalo-universitaire La Rabta, Jabbari, Tunis 1007, Tunisie; 4 Hôpital Européen Georges Pompidou, APHP, Unité de Parasitologie-Mycologie, Service de Microbiologie, 75015 Paris, France; 5 Université René Descartes, Faculté de médecine, 75006 Paris, France

**Keywords:** *Galleria mellonella*, *Aspergillus* spp., *Candida* spp., antifungal, pharmacokinetics

## Abstract

The treatment of invasive fungal infections remains challenging and the emergence of new fungal pathogens as well as the development of resistance to the main antifungal drugs highlight the need for novel therapeutic strategies. Although in vitro antifungal susceptibility testing has come of age, the proper evaluation of therapeutic efficacy of current or new antifungals is dependent on the use of animal models. Mammalian models, particularly using rodents, are the cornerstone for evaluation of antifungal efficacy, but are limited by increased costs and ethical considerations. To circumvent these limitations, alternative invertebrate models, such as *Galleria mellonella*, have been developed. Larvae of *G. mellonella* have been widely used for testing virulence of fungi and more recently have proven useful for evaluation of antifungal efficacy. This model is suitable for infection by different fungal pathogens including yeasts (*Candida*, *Cryptococcus*, *Trichosporon*) and filamentous fungi (*Aspergillus*, Mucorales). Antifungal efficacy may be easily estimated by fungal burden or mortality rate in infected and treated larvae. The aim of the present review is to summarize the actual data about the use of *G. mellonella* for testing the in vivo efficacy of licensed antifungal drugs, new drugs, and combination therapies.

## 1. Introduction

Animal models are still required for testing antifungal treatments prior to their use in humans. Experimental fungal infections are classically performed in rodents (mice, rats) or rabbits [1]. Despite their high relevance, small mammal models have significant drawbacks. In particular, they require dedicated infrastructures, they are difficult to implement, the durations of experimentation are usually long, and ethical considerations limit their use. For all these reasons, alternative models have been developed.

**Figure 1:**
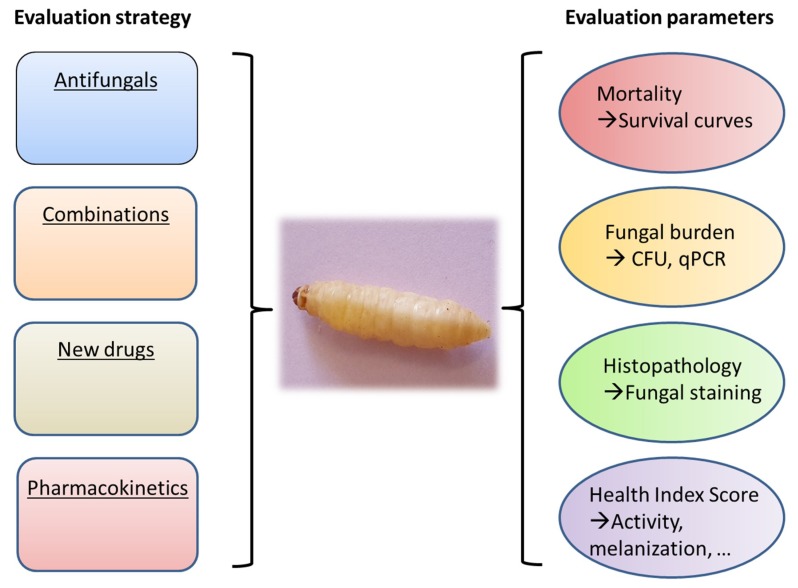
Role of *Galleria mellonella* for the in vivo evaluation of antifungals.

Several interesting mini-host models such as *Drosophila melanogaster*, *Caenorhabditis* elegans, and *Galleria mellonella* have been used for studying the pathophysiology of different fungal species [2,3] and more recently some antifungal treatments have also been evaluated in these models [4,5]. The *G. mellonella* model is particularly interesting because it is inexpensive, easy to use, and does not require specialized infrastructures. Larvae of the insect *G. mellonella* are small, allowing smooth handling, and can survive at 37 °C. The results of experiments are easy to be observed by the melanization of the larvae, decreased mobility, and death [6,7]. The immune system in *G. mellonella* is characterized by several types of hemocytes, several of which have the ability to neutralize and eliminate pathogens [8,9]. In medical mycology, this model has been used mainly for virulence studies but is now also used for antifungal evaluation [2,10,11] (Figure 1).

In this review, after a brief description of the methods and endpoints used for antifungal activity evaluation, we have summarized the actual data about the use of *G. mellonella* for testing the in vivo efficacy of antifungal drugs.

## 2. Methods and Endpoints Used for Evaluation of Antifungal Activity in *Galleria mellonella*

**Figure 2:**
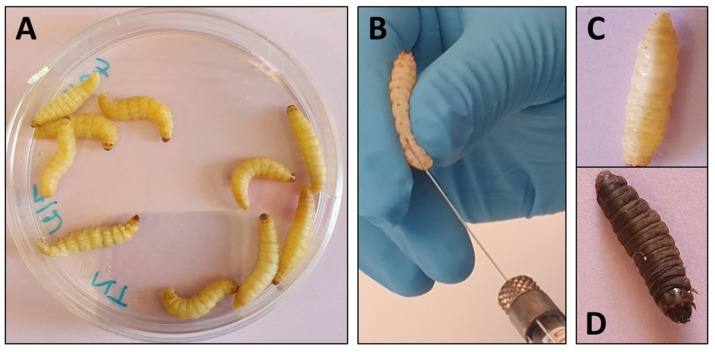
Use of *Galleria mellonella* larvae for evaluation of antifungal efficacy. (**A**) Larvae are grouped per ten in Petri dishes. (**B**) Inoculation and treatment are performed by injection in the ventral face of the last proleg with a Hamilton syringe. (**C**) Living larva. (**D**) Dead, melanized, larva.

*G. mellonella* larvae are on average 300 mg in weight and 2 cm in length which makes them easy to manipulate (Figure 2). Inoculations of larvae have essentially been performed by injection of a determined volume in ventral face of the last proleg by insulin or Hamilton syringe to reach the hemocoel [12]. The proleg region before injection has to be cleaned with 70% ethanol and larvae with dark spots or apparent melanization should be excluded. The number of larvae varies from 10 to 20 per groups and inoculation volume varies from 5 to 20 µl (10 µl in most cases) per larva. Preliminary experiments are generally needed to determine the lethal dose that gives 90% of mortality (LD90).

For treatment, the drugs are generally given by direct injection into the hemocoel. Most studies used a single treatment dose, but multiple dosing is also possible. Nevertheless, multiple injections may be traumatic, favor infection, and result in increased mortality. Among difficulties for treatment administration is the solvent used for drug solubilization. Indeed, many drugs need to be prepared in organic solvents such as dimethylsulfoxide (DMSO). Using DMSO is usually problematic, as this solvent is not so neutral for cell membranes, even at low concentrations, and may be toxic to larvae. This is not a problem for licensed antifungals for which the commercial preparations, which do not contain DMSO, are preferably used. Timing of antifungal dosing is an important parameter and should be optimized. Most often, curative treatment in which drugs are given 2–3 h after infection, have been used. Nevertheless, the efficacy of prophylactic treatment, in which drugs are given before infection can also be evaluated. Treatment with several drugs to assess the efficacy of combinations may also be performed. After inoculation with an LD90, larvae are randomly assigned to the different treatment groups. Two separate groups are given each monotherapy, while the combination group receives the two drugs which are generally given by separate injections. It has to be noticed that administration of some drugs (e.g., caspofungin) to larvae can trigger an immune response (immune priming) and one should be aware of this effect when interpreting results of antifungal efficacy.

Different endpoints may be used for evaluation of the efficacy of the antifungal treatment such as mortality, analysis of fungal burden and visualization of histological lesions in infected larvae after treatment (Figure 1). Mortality is recorded at least once daily, and survival data are used to generate Kaplan–Meier curves which can be analyzed by a log-rank test. Estimation of tissue fungal burden can be performed. After homogenization of larval tissues and appropriate dilutions, cultures are performed and Colony Forming Units (CFU) are determined. Use of quantitative PCR may be an alternative to cultures for estimation of fungal burden. Other outcome parameters may be used such as the health scoring system [7] that takes into accounts several endpoints (larval mobility, coccon formation, melanization, survival).

## 3. Evaluation of Antifungal Efficacy in *Galleria mellonella*

Antifungal efficacy has been evaluated in *G. mellonella* against a large panel of fungal species including, *Candida* spp. [13,14,15,16,17,18,19,20,21,22,23,24,25,26,27,28,29,30,31,32,33,34,35,36,37,38,39,40,41,42,43,44], *Cryptococcus* spp. [32,33,45,46,47,48,49,50,51], *Aspergillus* spp. [13,29,35,52,53,54,55,56,57,58,59,60,61], Mucorales [62,63,64], *Madurella mycetomatis* [65,66,67], and other fungal agents [68,69,70]. The contribution of *G. mellonella* to the evaluation of antifungal drugs will be detailed in the following paragraphs.

### 3.1. *Candida* spp.

The genus *Candida* has been extensively studied in the *G. mellonella* model especially for the evaluation of virulence and antifungal efficacy [10,71,72]. *G. mellonella* was used to test the efficacy of different antifungal compounds against *Candida* yeasts including conventional antifungal drugs [13,14,15,16,17,18,19,20,21,22,23], new drugs [25,26,27,30,31,32,34] or non-antifungal compounds in a repurposing perspective [24,28,29,33]. Antifungal combinations against *Candida* spp. have also been explored in the *G. mellonella* model [18,35,36,37,38,39,40,41,42,43,44,73].

#### 3.1.1. Conventional Antifungals

Several studies tested the efficacy of licensed antifungals (polyenes, azoles, echinocandins, and flucytosine) against different species of *Candida* (Table 1), such as *C. albicans* [13,14,17,18,21], *C. glabrata* [13], *C. krusei* [21], *C. tropicalis* [15,19], *C. parapsilosis* species complex [20,23], and uncommon *Candida* species [22].

Although *C. albicans* is the most frequent species involved in human infections, relatively few studies explored the use of *G. mellonella* for testing antifungal efficacy against this species. For example, Li et al. showed that fluconazole, amphotericin B, and flucytosine reduced mortality in a dose-dependent manner in larvae infected by *C. albicans* [18]. Fungal burden data were in concordance with survival results.

*C. glabrata* is the second cause of invasive candidiasis in USA and Central and Northern Europe [74]. Ames et al. [13] compared the efficacy of fluconazole, amphotericin B and caspofungin for the treatment of infected larvae by a fluconazole-susceptible strain of *C. albicans* and a strain of *C. glabrata* that displayed a high MIC of 32 µg/mL. Both strains were susceptible to caspofungin and amphotericin B. All dosages of fluconazole promoted survival whereas none protected against *C. glabrata* infection. Only the highest concentration of caspofungin or amphotericin B decreased the mortality of *C. glabrata* infected larvae while all doses of these drugs improved survival in *C. albicans*- infected larvae.

*C. tropicalis* is another common species evaluated in *G. mellonella* for the antifungal efficacy [15,19]. In a first study, it was shown that all the tested drugs (amphotericin B, caspofungin, fluconazole, and voriconazole) had a protective effect at clinically relevant doses [19]. In another study, several *C. tropicalis* isolates with different susceptibility profiles to fluconazole and voriconazole were used to infect larvae subsequently treated by fluconazole, voriconazole, amphotericin B, or anidulafungin [15]. Fluconazole improved survival in larvae infected with the fluconazole-susceptible isolate (MIC 0.5 µg/mL) but not in larvae infected by fluconazole-resistant isolates (MIC >64 µg/mL). Overall, there was a good correlation between the in vitro profile and the efficacy in the *G. mellonella* model. These studies demonstrated that *G. mellonella* was a good model for testing antifungal efficacy against *C. tropicalis*.

*C. krusei* is one of the most frequent *Candida* species with intrinsic resistance to fluconazole. Efficacy of fluconazole along with other antifungals (amphotericin B, voriconazole, and caspofungin) was evaluated in larvae infected by *C. krusei* and *C. albicans* [21]. Fluconazole did not protect larvae infected by *C. krusei* but improved survival in those infected by *C. albicans*. All other drugs improved survival during *C. krusei* infection but with lower efficacy than that observed during *C. albicans* infection showing that there was a good correlation between the in vitro susceptibility profile and efficacy in the mini model.

Two studies tested the in vivo efficacy of fluconazole against *C. parapsilosis* species complex isolates [20,23]. In the first study [23], one fluconazole-resistant isolate and one fluconazole- susceptible control isolate of *C. parapsilosis* were used to infect *G. mellonella* larvae. In terms of survival, fluconazole was active against the susceptible isolate but inactive against the resistant one and concordant results were obtained for the fungal burden. In the other study [20], a clinical isolate of *C. orthopsilosis* was found to be resistant to fluconazole due to a mutation G458S in Erg11. The in vitro resistance was confirmed in the *G. mellonella* model based on survival and fungal burden analysis.

Interestingly, *G. mellonella* was also used to evaluate the therapeutic efficacy in infections due to emerging *Candida* species. Silva et al. [22] compared the virulence of three species belonging to *C. haemulonii* complex and the efficacy of antifungal drugs in *G. mellonella* with non *albicans Candida* species. All clinical isolates of *C. haemulonii* species complex were resistant to azoles or amphotericin B and susceptible to caspofungin and these in vitro results were correlated with in vivo antifungal efficacy in *G. mellonella*. Indeed, only caspofungin had a protective effect, based on survival and fungal burden, in *C. haemulonii* infected larvae when compared with the untreated group. Again, this work showed the correlation between in vitro susceptibility profiles and in vivo efficacy of therapeutic doses in *G. mellonella*.

Beside the evaluation of antifungal activity against different species, the *G. mellonella* model can also be used to address unanswered issues. In that perspective, a recent study used the mini-model to explore the in vivo response to antifungals for isolates showing particular in vitro phenotypical characteristics such as trailing growth and paradoxical effect [14]. After selection of several *C. albicans* isolates exhibiting trailing to azoles and paradoxical effect with echinocandins, infected larvae were treated by voriconazole and caspofungin, respectively. Efficacy of voriconazole was poor in both susceptible or trailer isolates and the efficacy of caspofungin was variable among isolates showing in vitro paradoxical effect, precluding solid correlations between trailing or paradoxical effect and in vivo resistance.

Echinocandins are very potent antifungals and represent the first-line therapy for invasive candidiasis. It has been shown that in addition to their direct antifungal activity, echinocandins also possess immunomodulatory effects. Two studies used *G. mellonella* to demonstrate this effect [16,17]. In the study by Fuchs et al., caspofungin was able to increase survival in *C. albicans*-infected larvae but the drug also stimulated the innate immune response. This non-specific action protected larvae from other non-fungal microorganisms such as *Staphylococcus aureus*, which is not susceptible to the drug [17]. Similarly, another study showed that micafungin has an immunomodulatory effect in *G. mellonella*. Micafungin prophylaxis was able to protect larvae from bacterial infection [16]. It was shown that micafungin was able to increase hemocyte density in hemolymph.

#### 3.1.2. Combinations

Combination therapy is an interesting approach in difficult-to-treat infections. Antifungal agents may be combined with other antifungals but also with non-antifungal drugs. Several studies have used *G. mellonella* to demonstrate or to confirm synergistic interactions between drugs (Table 2).

For instance, the in vivo synergy between amphotericin B and flucytosine was demonstrated against *C. albicans* [18]. While monotherapy by amphotericin B or flucytosine was not effective, the combination of the two drugs significantly improved survival of infected larvae. Combination of amphotericin B with flucytosine is the cornerstone therapy for cryptococcosis and also used for difficult-to-treat invasive candidiasis such as endocarditis, endophthalmitis, and meningitis [76]. This combination may also be of interest for emerging *Candida* spp. such as *C. auris* [77] and *G. mellonella* will be an important tool to confirm the in vitro data.

Combination of antifungals with antibacterial drugs (Table 2) have also been explored [36,41,42,75]. For example, the synergy between linezolid, an oxazolidinone synthetic antibacterial, and azoles was evaluated against *C. albicans* [41]. In vitro combination of linezolid with azoles (fluconazole, itraconazole, or voriconazole) showed synergistic interactions against fluconazole- resistant *C. albicans*. In *G. mellonella*, only 20% of infected and untreated larvae survived four days after infection. After treatment with combination, survivals were 85%, 75%, and 80% for animals treated by linezolid+fluconazole, linezolid+itraconazole, and linezolid+voriconazole, respectively while it was only 25%, 40%, 35%, and 35% after monotherapy by linezolid, fluconazole, itraconazole, and voriconazole, respectively. In the study by Lu et al. it was shown that the combination of gentamicin, an aminoglycoside, with fluconazole was more effective than fluconazole alone in larvae infected with azole-resistant *C. albicans* both in term of survival and fungal burden [42]. These results confirmed the synergistic interactions between fluconazole and gentamicin found in vitro. The in vivo efficacy of tetracycline antibiotics in combination with fluconazole has also been tested. Minocycline+fluconazole and doxycycline+fluconazole combinations in *G. mellonella* were tested by survival analysis, quantification of *C. albicans* CFU/mL and histological analysis [36]. It was concluded that the combinations were synergistic although the level of significance was not reported. In another study, the synergistic effect of colistin (and other antimicrobial peptides) combined with caspofungin was demonstrated in vivo, confirming the in vitro data [75]. There is no doubt that the *G. mellonella* model will be further useful for the in vivo evaluation of promising combinations between antifungals and antibiotics against emerging fungal pathogens such as *C. auris* [78].

Several other studies (Table 2) used *G. mellonella* to demonstrate synergistic interactions between antifungals (mainly fluconazole) and other drugs against *C. albicans* [35,37,38,39,40,43,44,73]. The partner drugs were anti-inflammatory compounds such as dexamethasone [43] and licofelone [40], D- penicillamine, a heavy metal chelator [39], harmine, an alkaloid with multiple pharmacological properties [37], ambroxol, a mucolytic drug [38], antivirals such as ribavirin [44], proton-pump inhibitors [73], or Hsp-90 (a molecular chaperone implicated in cellular response to stress) inhibitors [35].

#### 3.1.3. New Drugs

Several studies which have focused on the development of new drugs have benefited from the use of *G. mellonella* as a model for evaluating in vivo efficacy. Several drugs, such as atorvastatin, a cholesterol-lowering agent [24], miltefosine, an anti-leishmanial drug [33], miramistin, an antiseptic [29], and pilocarpine, a muscarinic agonist [28] were evaluated in a repurposing perspective. In other instances, new synthetic compounds or natural products were tested in vivo in the model [25,26,27,30,31,32,34]. It must be noticed that in all cases, larvae were infected with *C. albicans*.

The large amount of currently available data shows that *G. mellonella* is a very interesting model to evaluate the in vivo efficacy of drugs against *Candida* spp.

### 3.2. *Cryptococcus* spp.

The interest and the contribution of *G. mellonella* for the evaluation of the virulence of *Cryptococcus neoformans* and its susceptibility to antifungal drugs was demonstrated fifteen years ago [47]. It was shown that *G. mellonella* is a valuable model due to its thermotolerance and its high susceptibility to infection unlike other invertebrates, like adults of *Drosophila melanogaster* which are resistant to *C. neoformans* [79]. A summary of studies using *G. mellonella* as a model for antifungal evaluation against *Cryptococcus* spp. is shown in Table 3.

In what is probably the first study of antifungal efficacy in *G. mellonella*, Mylonakis et al. [47] used the conventional antifungal agents for the treatment of *Cryptococcus* infection. The administration of amphotericin B or flucytosine was effective in reducing mortality compared to untreated controls. Fluconazole seemed also to be effective, although statistically significance was not reached. It was also shown that the combination of amphotericin B with flucytosine significantly decreased the mortality of infected larvae compared to amphotericin B alone.

Among other licensed antifungals, voriconazole was more recently tested for efficacy against *C. neoformans* and *C. gattii* infection in *G. mellonella* [45]. Voriconazole treatment significantly increased survival and decreased fungal burden in infected larvae by both species.

Drug repurposing is currently a widely explored strategy for treatment optimization of cryptococcosis. In that perspective, several compounds such as miltefosine and astemizole, have been tested in *G. mellonella* for their efficacy against cryptococcosis [33,51]. The anti-leishmanial miltefosine, which is also known to possess antifungal activity, was evaluated for the treatment of cryptococcosis. Treatment of *G. mellonella* larvae with miltefosine embedded in alginate nanoparticles reduced mortality and fungal burden in *C. gattii*-infected larvae [33]. Similarly, astemizole, an antihistaminic drug, as well as one of its analogues were tested in combination with fluconazole [51]. The combination was fungicidal in vitro and significantly increased survival of *G. mellonella* larvae infected by a fluconazole-susceptible *C. neoformans* isolate.

The potential use of *G. mellonella* for evaluation of treatment efficacy has also been highlighted in several studies that focused on the development of new molecules with antifungal activities against *Cryptococcus* spp. [32,46,48,49,50].

Among these studies, Sa et al. [32] evaluated a phenylthiazole derivative (CHT) for the treatment of cryptococcosis and candidiasis in animal models (*G. mellonella* and a murine model). CHT treatment significantly prolonged survival in *C. albicans*, *C. gattii* or *C. neoformans*-infected larvae and the results were confirmed in a murine model. In another study from the same group [46], the effect of a closely related thiazole derivative (named compound 3) was evaluated for the treatment of *C. neoformans* or *C. gattii*-infected larvae. Treatment with compound 3 resulted in increased survival of infected larvae with an efficacy similar to that of fluconazole.

Sometimes, in vitro activity is not translated to in vivo efficacy in *G. mellonella*. For example, Palanco et al. [48] tested the efficacy of 3′-hydroxychalcone. Chalcones, which are abundant in plants, have numerous pharmacological activities including antifungal action [80]. The efficacy of this compound was evaluated on groups of larvae infected by one isolate of *C. gattii* (collected from a psittacine bird) or the strain *C. gattii* ATCC 56990 (collected from a human). In vitro, 3′hydroxychalone was fungicidal against *C. gattii* both in planktonic and biofilm form but did not decrease the mortality or the fungal burden of infected larvae. This lack of correlation between in vitro activity and in vivo efficacy could possibly be explained by the high hydrophobicity of the compound responsible for a limited distribution in tissues [48].

Other natural substances, such as pedalitin, a compound isolated from a plant, were tested against *C. neoformans* both in vitro and in vivo by using *G. mellonella* larvae [49]. The combination of pedalitin with amphotericin B was synergistic in vitro. By evaluating mortality, fungal burden, and histopathology, the authors showed that the combination was also effective in vivo in *G. mellonella*, and that there was a good correlation with the results obtained in the murine model.

Beside plants, animals are also a source of natural compounds with antimicrobial activity. In this context, an antimicrobial peptide analogue isolated from the wasp venom was tested for its anti- cryptococcal activity [50]. In this study the peptide was active in vitro against *C. neoformans*, had low toxicity toward mammalians cells, and increased survival in a *G. mellonella* model of cryptococcosis. The combination of this peptide with either amphotericin B or fluconazole was not more effective than monotherapies.

To conclude, *G. mellonella* is a suitable model for *Cryptococcus* infection and could be used for evaluation of antifungal treatments in cryptococcosis. It is interesting to note that very different drugs, including licensed antifungals, new synthetic molecules, or natural compounds, may be evaluated in the *G. mellonella* model.

### 3.3. *Trichosporon* spp.

*Trichosporon* yeasts are responsible for superficial infections but may also behave as opportunistic agents of invasive infections mainly in patients with hematological malignancies [81]. The incidence of fungemia caused by *Trichosporon* spp. is increasing in patients with hematological malignancies and neutropenia and traditional antifungal drugs are not very efficient against *Trichosporon* spp. [82]. In a recent study, *G. mellonella* was evaluated as an animal model of *Trichosporon* spp. infection [68]. The authors compared the susceptibility of *Trichosporon* strains to three antifungals drugs in vitro and in vivo in two animal models (mice and *G. mellonella*). Immunocompromised mice and *G. mellonella* larvae were infected by different strains of *T. asahii* (n = 3)*, T. asteroids* (n = 3), or *T. inkin* (n = 1) and treated with amphotericin B or azoles. Fluconazole was able to improve the survival in both animal models against the three *Trichosporon* species. In contrast, voriconazole was more effective in the *G. mellonella* model, possibly due to the rapid metabolism of this drug in mice. Amphotericin B was not able to reduce mortality in any cases in infected larvae but only in mice infected with *T. asahii* strains.

Overall, it can be concluded that the *G. mellonella* model could be useful for the evaluation of antifungal treatments against *Trichosporon* spp.

### 3.4. *Aspergillus* spp.

The treatment of *G. mellonella* infected with *Aspergillus* spp. has been evaluated in several studies (Table 4) in order to assess intrinsic resistance in specific species [55,56,83], acquired azole resistance in *A. fumigatus* [53,54], combinations [35,59], and efficacy of non-antifungal drugs [29,57,58,60,61].

Susceptibility to azoles in cryptic *Aspergillus* species differs. *Aspergillus lentulus*, a cryptic species of the *A. fumigatus* complex (*Aspergillus* section *Fumigati*) is less susceptible to voriconazole [84]. Alcazar-Fuoli et al. [83] compared the efficacy of voriconazole on larvae infected with *A. fumigatus* sensu stricto or with *A. lentulus*. When *A. fumigatus* was used, 100% of infected larvae died four days after infection. However, only 80% of larvae died by day 10 after infection by *A. lentulus*. Treatment with voriconazole resulted in a significant decrease in mortality in larvae infected with *A. fumigatus* compared to untreated controls but not in larvae infected by *A. lentulus*. Species other than *A. fumigatus* are emerging and are characterized by a decreased susceptibility to azoles. Glampedakis et al. [55] evaluated different antifungals alone or in combination against *A. calidoustus* both in vitro and in vivo in the *G. mellonella* model. Although amphotericin B showed a better in vitro activity, it was not more active than voriconazole in vivo. Voriconazole combined with terbinafine also showed potential benefit. Susceptibility of *Aspergillus terreus* to amphotericin B has been evaluated in vitro and in vivo by Maurer et al. [56] by testing susceptible and resistant isolates. Treatment of infected larvae with liposomal-amphotericin B reduced mortality only in larvae infected with the susceptible isolates, demonstrating a good correlation between the in vitro results and the in vivo efficacy in *G. mellonella*. The model was subsequently used to demonstrate that Hsp70 proteins play an important role in the response of *A. terreus* to amphotericin B [52]. Against amphotericin B resistant strains of *A. terreus*, the combination of amphotericin B and an Hsp70 inhibitor increase survival of larvae compared to amphotericin B alone.

Beside intrinsic resistance, acquired resistance in *A. fumigatus* has become a major problem. Indeed, the last decades have been marked by the emergence of azole-resistant strains of *A. fumigatus* linked to long-term antifungal treatment in patients and fungicide use in the environment [85]. Forestiero et al. [53] tested the efficacy of different dosages of voriconazole and posaconazole on larvae infected by a wild type of *A. fumigatus* sensu stricto or different isolates with Cyp51A mutations such as G54W or TR/L98H conferring high MICs to posaconazole and/or voriconazole. For larvae infected with azole susceptible strains of *A. fumigatus* (voriconazole MIC ≤1 mg/L) and treated with voriconazole, the median survival time was 2 to 3 days versus 7 days in untreated larvae. In contrast, in larvae infected with resistant strains (voriconazole MIC of 4 mg/L), survival was not statistically different between treated larvae and untreated controls. In another study, *Aspergillus fumigatus* isolates with single nucleotide polymorphism (SNP) in Cyp51A and low level of resistance (higher azole MICs but not categorized as resistant based on the current clinical breakpoints) were evaluated in vivo to test if the SNPs were associated with a poorer treatment response. Voriconazole treatment improved survival of larvae infected with a wild-type isolate (40% survival). Isolates with Cyp51A SNPs also responded to voriconazole to a certain extent but showed a 0% survival [54].

Up to now, few studies have tested combination treatment of aspergillosis in the *G. mellonella* model. In one study, combination of geldanamycin, an inhibitor of Hsp 90, with caspofungin was tested in vitro and in vivo against *A. fumigatus*. While caspofungin or geldanamycin alone did not increase survival of larvae inoculated with a lethal dose, the combination of the two drugs significantly protected larvae from death [35]. In the same way it was shown that the combination of itraconazole with EGTA (ethylene glycol tetra-acetic acid), a calcium chelator, was synergistic [59]. Larvae inoculated by a lethal dose of *A. fumigatus* had a survival of 60% when treated with itraconazole alone, and 87% when itraconazole was combined with EGTA.

*G. mellonella* has also been used as an in vivo model for the evaluation of antifungal efficacy of new drugs [57,58,60] or in the context of repurposing [29,61]. By screening large chemical libraries, several drugs with promising antifungal activity were identified and further tested in animal models, including *G. mellonella* [57,58,60]. Bromoquinol, a quinoline, showed efficacy similar to that of the amphotericin B used as a positive control [58]. Nevertheless, the compound showed lower efficacy in a murine model of pulmonary aspergillosis. While amphotericin B was effective compared to controls, bromoquinol did not significantly improve survival of infected mice. Another compound with activity at the cell wall level, called haemafungin, was tested in *G. mellonella* for its antifungal efficacy [57]. In larvae inoculated with a lethal dose of *A. fumigatus*, haemofungin had a similar efficacy to amphotericin B. A new class of compounds based on a 4-chloro-6-arylamino-7-nitro-benzofurazane molecular structure (CANBEFs) has been identified as potential antifungals [60]. Although one of the compounds (CANBEF-24) showed promising in vitro activity, it was not effective in vivo. Sertraline, a selective serotonin reuptake inhibitor used as antidepressant also has antifungal activities and it has been used recently as an adjunctive therapy in cryptococcal meningitis [86]. Trevino-Rangel et al. [61] compared the efficacy of sertraline for the treatment of invasive aspergillosis in *G. mellonella* and BALB/c male mice to the efficacy of amphotericin B and voriconazole. Outcome parameters were survival in *G. mellonella* and fungal burden in mice. Results showed a survival of >50% after treatment by voriconazole or amphotericin B and 25% after treatment by sertraline. In accordance with the results obtained in the *G. mellonella* model, there was a significant reduction in the pulmonary fungal burden in infected mice treated by sertraline. Miramistin, a topical antiseptic, has been tested in vivo and showed efficacy in larvae infected with *A. fumigatus* [29].

*G. mellonella* is a suitable host for the development of *Aspergillus* spp. infection and a valuable alternative to mammalian models for testing the in vivo efficacy of antifungals against aspergillosis.

### 3.5. Mucorales

Mucorales are ubiquitous fungi that are responsible for difficult-to-treat fungal infections in diabetic and immunocompromised individuals [87,88,89]. Recent years have been marked by a significant increase in mucormycoses worldwide [88,90]. Growth at elevated temperatures is known to be an important virulence factor in several fungal pathogens including Mucorales [91]. Kaerger et al. [91] used *G. mellonella* to compare the virulence of five thermotolerant Mucorales species: *Rhizopus arrhizus, R. microsporus, R. homothallicus, R. caespitosus,* and *R. schipperae*. Maurer et al. [64] showed variability in the virulence of Mucorales as a function of temperature and *Rhizopus*-infected larvae died more quickly when they were incubated at 37 °C then at 30 °C.

Although several authors compared the virulence of Mucorales in *G. mellonella,* only three studies used this model to evaluate antifungal activity (Table 5). Maurer et al. [64] used *G. mellonella* to test the efficacy of liposomal amphotericin B, posaconazole, isavuconazole, and nystatin-intralipid, an antifungal formulation that has proven to be effective against invasive aspergillosis and invasive candidiasis in a murine model [92]. Antifungals were tested against six Mucorales species, *Lichtheimia corymbifera*, *L. ramosa, R. arrhizus, R. microsporus, Mucor circinelloides,* and *Rhizomucor pusillus*, and the efficacy was compared to in vitro susceptibility tests [64]. Nystatin-intralipid showed the better efficacy against all the species except *R. arrhizus*. Particularly, survival was improved by 60% and 30% in nystatin-intralipid treated larvae infected by *L. corymbifera* and *L. ramosa*, respectively. These results correlated with the high in vitro activity against *Lichtheimia* spp. Liposomal amphotericin B, which exhibited the better in vitro activity but showed a low in vivo efficacy except against *L. corymbifera*. Isavuconazole was not effective except in *M. circinelloides* infected larvae. The pharmacokinetics of isavuconazole, nystatin-intralipid, posaconazole and liposomal amphotericin B was also determined by bioassay in hemolymph at different time points after injection. The concentration of isavuconazole was below the MIC value at 6 h post injection and reached undetectable levels at 16 h. This could partly explain its limited efficacy. It has also to be noted that esterases are necessary to transform the pro-drug to active isavuconazole. Although esterase activity has been reported in *G. mellonella* [93] it is not yet known if such esterase activity is able to produce the active drug [64].

Macedo et al. [63] evaluated the efficacy of monotherapy based on therapeutic doses of voriconazole, amphotericin B, caspofungin, and posaconazole for the treatment of larvae infected by *R. microsporus* or *R. arrhizus*. Combinations of voriconazole with amphotericin B, caspofungin or posaconazole were also tested by using the lowest antifungal doses that gave hyphae alterations in vitro. Monotherapies did not improve survival of infected larvae except for caspofungin in *R. arrhizus*-infected larvae. Combination of voriconazole with amphotericin B, and voriconazole with caspofungin (despite the reduction of doses of drugs in association) improved the median survival in *R. microsporus*-infected larvae but not in *R. arrhizus*-infected larvae compared to monotherapy. Non-antifungal drugs have also been tested against Mucorales in *G. mellonella*. For example, rapamycin [62] improved survival by 50% in *M. circinelloides*-infected larvae.

Although *G. mellonella* was successfully used to study virulence of Mucorales, more studies are necessary to confirm its usefulness for in vivo evaluation of antifungal treatments.

### 3.6. Madurella Mycetomatis

Eumycetoma are tropical infections due to different fungal species including *Madurella mycetomatis*. For these infections, there is a limited efficacy of antifungal treatment probably related to the ability of the causative fungi to form grains in infected tissues. Inside the grains, hyphae are protected by a barrier that reduces the action of the antifungal. Because eumycetomas are rare diseases, clinical trials are difficult to perform, and animal models may be of great value for the evaluation of new therapeutic strategies. By comparing histological sections of *G. mellonella* larvae infected with *M. mycetomatis* to those of patients and mice, it was demonstrated that grains produced in the larvae resembled those formed in mammalian hosts [94].

The interest of testing the efficacy of antifungals in vivo and more precisely in *G. mellonella* was highlighted by Kloezen et al. [66]. The authors used a prophylactic (2-h before infection) or a curative (4-h after infection when grains are already formed) treatment to compare the efficacy of the antifungals. They showed that only amphotericin B and terbinafine increased the survival of *M. mycetomatis*-infected larvae whether the protocol was prophylactic or curative. In contrast, azoles did not improve survival. Antifungal combinations were also tested in the same model [65]. Combination of itraconazole with terbinafine was not beneficial, while combination of amphotericin B with either itraconazole or terbinafine decreased survival compared to amphotericin B alone and was therefore antagonistic.

*G. mellonella* has also been used to test new drugs against *M. mycetomatis* [67]. After the in vitro screening of 800 molecules, the 10 most potent compounds were tested in vivo in *G. mellonella*. Several of these compounds showed activity in term of prolonged survival and reduced fungal burden. In particular, a fenarimol analogue, a non-azole Cyp51 inhibitor, showed promising results. Based on these results, several other fenarimol analogues were also tested in *G. mellonella* model [67].

Overall, several studies have demonstrated that *G. mellonella* is a suitable model for *M. mycetomatis* infection with the formation of typical fungal grains in larvae tissue. Moreover, the model seems to be valuable for assessing antifungal efficacy and screening of new molecules potentially active against this difficult-to-treat infection.

## 4. Pharmacokinetics of Antifungals in *G. mellonella*

The purpose of in vivo experimentation in animal models is essentially the ability to extrapolate the results to human. For this, the pharmacokinetics of antifungals in the animal models used for evaluating antifungal efficacy must be known. The main parameters are the maximum concentration (C_max_), the area under the curve (AUC), and the half-life (T_1/2_). In *G. mellonella*, several studies evaluated the pharmacokinetics of different drugs including ketoconazole, fluconazole, voriconazole, posaconazole, isavuconazole, amphotericin B, nystatin intra-lipid, and terbinafine [53,64,66,95].

Astvad et al. [95] described the pharmacokinetics of fluconazole in *G. mellonella* hemolymph and tried to determine a humanized dose. The pharmacokinetics of fluconazole showed a linear increase in C_max_ and AUC_0-24_ with the dose. Despite the lack of kidneys, the clearance of the drug in larvae was much greater than in humans and the T_1/2_ was about one third of the human value. Overall, the authors found that a dose of 20 mg/kg of larval tissues would result in a similar exposure to that obtained in humans with a standard dose of fluconazole.

In another study, the pharmacokinetic parameters of voriconazole and posaconazole were determined in non-infected *G. mellonella* larvae [53]. AUC_0-24_ of voriconazole after a single dose of 10 mg/kg was in the same range as the value observed in human subjects receiving a dose of 8 mg/kg/day. Similarly, for posaconazole, exposure (AUC_0-24_) was similar in *G. mellonella* treated by a single injection of 10 mg/kg and in humans treated by 800 mg/day.

Maurer et al. [64] determined the pharmacokinetics of amphotericin B, isavuconazole, posaconazole, and nystatin intra-lipid after a 15 mg/kg single dose administration in *G. mellonella* larvae. They showed that the polyenes were more stable in the hemolymph than azoles and that they exhibited higher T_1/2_ and AUC values. The hemolymph concentration of posaconazole and isavuconazole reached an undetectable level at 22 h and 16 h after administration, respectively.

In another study, pharmacokinetic characteristics of four azoles (ketoconazole, itraconazole, voriconazole, and posaconazole), amphotericin B, and terbinafine were determined [66]. For amphotericin B given at 1 mg/kg, C_max_ was comparable in *G. mellonella* and in humans. For azoles, all given at 5.7 mg/kg, different results were obtained: compared to humans, AUC in *G. mellonella* was lower for ketoconazole and itraconazole, similar for posaconazole, and higher for voriconazole.

Overall, the available studies showed that antifungal drugs are distributed and eliminated in *G. mellonella* larvae and that it is possible, at least for some antifungal drugs, to use doses that allow exposure similar to that observed in humans. Most of the pharmacokinetic data were obtained in non-infected larvae. Therefore, further studies are warranted to explore possible alterations of pharmacokinetic parameters in infected animals.

## 5. Conclusions and Perspectives

Although *G. mellonella* was primarily used to study virulence, it is now widely used for testing drugs’ efficacy against different fungal species. In many instances, a good correlation with in vitro results and mammalian models has been obtained. Therefore, *G. mellonella* represents an alternative to mammalian models as a screening tool for antifungal evaluation. The main advantages are the rapidity and low costs compared to the standard mammalian models.

Further studies are needed to refine the reproducibility and standardization of the models developed in *G. mellonella*. More fungal species must be tested and pharmacokinetic data, particularly in infected larvae, need to be further evaluated.

## Figures and Tables

**Table 1:** Evaluation of licensed antifungals efficacy against *Candida* spp. in *G. mellonella*.

Species	Antifungal	Dosage In Vivo (mg/kg)	In Vitro Phenotype	In vivo Efficacy (Gm)	Reference
*C. albicans*	FCZ	3, 6, 12	S	Yes	[13]
*C. albicans*	AMB	2, 4	S	Yes	[13]
*C. albicans*	CAS	1, 2, 4	S	Yes	[13]
*C. albicans*	FCZ	1, 4, 16	S	Yes	[18]
*C. albicans*	FCZ	4	R	No	[18]
*C. albicans*	AMB	0.4, 1.6, 6.4	S	Yes	[18]
*C. albicans*	5FC	1.25, 5, 20	S	Yes	[18]
*C. albicans*	AMB	1, 2, 4	S	Yes	[21]
*C. albicans*	FCZ	4, 12, 32, 64	S	Yes	[21]
*C. albicans*	VRZ	7.5, 10	S	Yes	[21]
*C. albicans*	CAS	1, 2, 4	S	Yes	[21]
*C. glabrata*	FCZ	3, 6, 12	32	No	[13]
*C. glabrata*	AMB	2, 4	S	No at 2, Yes at 4	[13]
*C. glabrata*	5FC	1, 2, 4	S	No at 1, Yes at 2 and 4	[13]
*C. tropicalis*	FCZ	9	S	Yes	[15]
*C. tropicalis*	FCZ	9	R	No	[15]
*C. tropicalis*	VRZ	10	S	Yes	[15]
*C. tropicalis*	VRZ	10	R	No	[15]
*C. tropicalis*	AMB	3	S	Yes	[15]
*C. tropicalis*	AMB	3	R	No	[15]
*C. tropicalis*	ANI	10	S	Yes	[15]
*C. tropicalis*	AMB	1, 2, 4	S	Yes	[19]
*C. tropicalis*	FCZ	ND	S	Yes high dose, No low dose	[19]
*C. tropicalis*	VRZ	ND	S	Yes high dose, No low dose	[19]
*C. tropicalis*	CAS	1, 2, 4	S	Yes	[19]
*C. krusei*	AMB	1, 2, 4	S	No at 1 and 2, Yes at 4	[21]
*C. krusei*	FCZ	4, 12, 32, 64	R	No	[21]
*C. krusei*	VRZ	7.5, 10	S	No at 7.5, Yes at 10	[21]
*C. krusei*	CAS	1, 2, 4	S	No at 1 and 2, Yes at 4	[21]
*C. orthopsilosis*	FCZ	2, 10	S	Yes	[20]
*C. orthopsilosis*	FCZ	2, 10	R	No	[20]
*C. parapsilosis*	FCZ	14	S	Yes	[23]
*C. parapsilosis*	FCZ	14	R	No	[23]
*C. haemulonii*	FCZ	6, 12	R	No	[22]
*C. haemulonii*	AMB	2.5, 5	R	No	[22]
*C. haemulonii*	CAS	0,5, 1	S	Yes	[22]
*C. duobushaemulonii*	FCZ	6, 12	R	No	[22]
*C. duobushaemulonii*	AMB	2.5, 5	R	No	[22]
*C. duobushaemulonii*	CAS	0,5, 1	S	Yes	[22]
*C. tropicalis*	FCZ	6, 12	S	Yes	[22]
*C. tropicalis*	AMB	2.5, 5	S	Yes	[22]
*C. tropicalis*	CAS	0,5, 1	S	Yes	[22]
*C. krusei*	FCZ	6, 12	R	No	[22]
*C. krusei*	AMB	2.5, 5	S	Yes	[22]
*C. krusei*	CAS	0,5, 1	S	Yes	[22]
*C. lusitaniae*	FCZ	6, 12	S	Yes	[22]
*C. lusitaniae*	AMB	2.5, 5	R	No at 2.5, Yes at 5	[22]
*C. lusitaniae*	CAS	0,5, 1	S	Yes	[22]

AMB: amphotericin B; VRZ: voriconazole; FCZ: fluconazole; 5FC: flucytosine; CAS: caspofungin; ANI: anidulafungin; Gm: *Galleria mellonella.* R: resistant; S: susceptible.

**Table 2:** Evaluation of antifungal combination efficacy against *Candida* spp. in *G. mellonella.*

Species	Drugs in Combination		Efficacy of the Combination		Reference
	Partner #1	Partner #2	In Vitro (SYN)	In Vivo (Gm)	
*C. albicans*	AMB	5FC	ND	Yes	[18]
*C. albicans* (Razole)	FCZ	Linezolid	Yes	Yes	[41]
*C. albicans* (Razole)	ITZ	Linezolid	Yes	Yes	[41]
*C. albicans* (Razole)	VRZ	Linezolid	Yes	Yes	[41]
*C. albicans* (Razole)	FCZ	Gentamicin	Yes	Yes	[42]
*C. albicans* (Razole)	FCZ	Minocycline	ND	Yes	[36]
*C. albicans* (Razole)	FCZ	Doxycycline	ND	Yes	[36]
*C. albicans*	CAS	Colistin	Yes	Yes	[75]
*C. albicans* (Razole)	FCZ	Dexamethasone	Yes	Yes	[43]
*C. albicans* (Razole)	FCZ	Licofelone	Yes	Yes	[59]
*C. albicans* (Razole)	FCZ	D-penicillamine	Yes	Yes	[39]
*C. albicans* (Razole)	FCZ	Harmine	Yes	Yes	[37]
*C. albicans* (Razole)	FCZ	Ambroxol	Yes	Yes	[38]
*C. albicans* (Razole)	FCZ	Ribavirin	Yes	Yes	[44]
*C. albicans* (Razole)	FCZ	Proton-pump inhibitors	Yes	Yes	[73]
*C. albicans*	FCZ	Hsp90 inhibitors	Yes	Yes	[35]

Razole: strain resistant to azole drugs; AMB: amphotericin B; ITZ: itraconazole; VRZ: voriconazole; FCZ: fluconazole; 5FC: flucytosine; CAS: caspofungin; SYN: synergy; Gm: *Galleria mellonella*.

**Table 3:** Evaluation of antifungal activity for treatment of *Cryptococcus* infection in *Galleria mellonella*.

Species	Antifungals (doses [mg/kg])	Combination	Main Results	Reference
*C. neoformans*	AMB (1.5) FCZ (14) 5-FC (20)	Yes	AMB or FC alone prolonged survival, FCZ prolonged survival (NS) AMB+FC more effective then AMB alone	[47]
*C. neoformans* *C. gattii*	VRZ (10, 20) AMB (1, 10, 20)	No	VRZ increased survival and decreased fungal burden	[45]
*C. neoformans* *C. gattii*	MFS*	No	MFS increased survival for *C. gattii*, and decreased fungal burden for both species	[33]
*C. neoformans*	AST, A2 FCZ	Yes	FCZ+AST and FCZ+A2 increased survival in larvae infected with FCZ-susceptible isolate	[51]
*C. gattii*	3′-hydroxychalcone (2, 80, 160) AMB (2)	No	No in vitro–in vivo correlation. 3- hydroxychalcone fungicidal in vitro but no efficacy in vivo in terms of survival and fungal burden	[48]
*C. gattii* *C. neoformans*	CHT (5, 10) FCZ (10)	No	CHT increased survival for *C. gattii* and *C. neoformans* infected larvae. Correlation with a murine model	[32]
*C. neoformans* *C. gattii*	Compound 3 (5, 10) FCZ (5)	No	Compound 3 increased survival of infected larvae. Efficacy similar to that of FCZ	[46]
*C. neoformans*	PED (6.25 to 200) AMB (0.5 to 4)	Yes	AMB or PED increased survival. Better efficacy of the combination Good correlation with the murine model	[49]
*C. neoformans*	MK58911 (10 to 100) AMB (4) FCZ (10)	Yes	MK58911 increased survival. No benefit of MK58911+ AMB and MK58911+FCZ compared to monotherapies	[50]

AMB: amphotericin B; FCZ: fluconazole; FC: flucytosine; CHT: 2-(2-(cyclohexylmethylene)hydrazinyl)-4-phenylthiazole; compound 3: 2-[2-(cyclohexylmethylene)hydrazinyl)]-4-)4-methoxyphenyl) thiazole; PED: pedalitine; MFS: miltefosine (free miltefosine (10 to 40 mg/kg) or miltefosine-loaded alginate nanoparticles (100 or 200 mg/kg)); AST: astemizole; A2: astemizole analogue #2 (1H-Benzimidazole-2-amine,1-[2-(4-methoxyphenyl)ethyl]-N-[1-[2-(4-methoxyphenyl)ethyl]-4-piperidinyl]; NS: Not Significant.

**Table 4:** Evaluation of antifungal treatment against *Aspergillus* spp. in *Galleria mellonella*.

Species	Antifungals (doses [mg/kg])	Combination	Main Results	Reference
*A. lentulus*	VRZ (10)	No	No efficacy of VRZ against *A. lentulus* (azole-resistant) compared to *A. fumigatus* in term of survival and fungal burden	[83]
*A. calidoustus*	VRZ (10) AMB (5) TBF (5)	Yes	AMB not superior to VRZ in vivo, in contrast to in vitro. TBF combined with VRZ better than monotherapies. Combination synergistic in vitro.	[55]
*A. terreus*	L-AMB (1.6, 16.6)	No	Efficacy of L-AMB against AMB-susceptible isolates and no efficacy against AMB-resistant isolates. L-AMB administration increased hemocyte density.	[56]
*A. terreus*	AMB (5) Hsp70 inhibitor	Yes	AMB+Hsp70 inhibitor decreased MIC in vitro and increased survival in larvae infected with AMB-resistant isolate	[52]
*A. fumigatus*	VRZ (1.25, 2.5, 10, 40, 80) PSZ	No	VRZ at 10 mg/kg improved survival against VRC-susceptible strains (MIC ≤ 1 mg/L) but not against VRZ-resistant strains (MIC = 4 mg/L).	[53]
*A. fumigatus*	VRZ (10)	No	VRZ increased survival of larvae infected by either WT and mutant (isolates with SNPs in CYP51A and moderately elevated MICs) although mortality rate was higher for mutants.	[54]
*A. fumigatus*	CAS (1.5) GdA	Yes	Combination therapy (GdA + CAS) improved survival compared to each monotherapy. Correlation with in vitro results	[35]
*A. fumigatus*	ITZ (100) EGTA	Yes	The calcium chelator EGTA was synergistic in vivo when combined with ITZ.	[59]
*A. fumigatus*	AMB (2) Haemofungin	No	*In vitro* (CLSI), combination HMG+AMB not synergistic in contrast to HMG+CAS Efficacy of haemofungin (5.7 mg/kg) similar to that of amphotericin B at 2 mg/kg	[57]
*A. fumigatus*	AMB (1, 2) BMQ	No	Similar efficacy of BMQ (8 mg/kg) and AMB in *G. mellonella*. No correlation with a murine model.	[58]
*A. fumigatus*	CANBEF-24 (1.8, to 14.4)	No	No in vivo efficacy despite in vitro activity.	[60]
*A. fumigatus*	Miramistin	No	In vivo efficacy of miramistin	[29]
*A. fumigatus*	VRZ (10) AMB (3) sertraline (3, 10)	No	Survival of 50% for AMB and VRZ and 25% for sertraline. Correlation with murine model.	[61]

AMB: amphotericin B, VRZ: voriconazole, TBF: terbinafine, CAS: caspofungin, BMQ: bromoquinol, CANBEF-24: 4-chloro-6-arylamino-7-nitro-benzofurazane, GdA: geldanamycin, Gm: *Galleria mellonella*.

**Table 5:** Evaluation of antifungal treatment against Mucorales in *Galleria mellonella*.

Species	Antifungals (Doses [mg/kg])	Combination	Main Results	Reference
**6 species^a^**	AMB (1), VRZ (10), CAS (0.5), PSZ (10)	Yes	No efficacy of monotherapies except for CAS vs. *R. microsporus* VRZ+AMB and VRZ+CAS increased survival compared to AMB alone for *R. microsporus* but not for *R. oryzae*	[63]
**6 species^b^**	AMB (15), CAS (15), PSZ (15), NYS-L (15)	No	NYS-L has the best efficacy except for *R. arrhizus* L-AMB has low efficacy except for *L. corymbifera* ISA not effective except for *M. circinelloides*	[64]
***M. circinelloides***	Rapamycin (33)	No	Rapamycin increased survival	[62]

^a^ R. microsporus, R. oryzae, S. racemosum, Lichtheimia corymbifera, L. blaskesleeana, L. ramosa. ^b^ R. arrhizus, R. microsporus, L. corymbifera, L. ramosa, M. circinelloides, Rh. Pusillus. AMB: amphotericin B; VRZ: voriconazole; CAS: caspofungin; PSZ: posaconazole; NYS-L: nystatin intralipid; L-AMB: liposomal amphotericin B; ISA: isavuconazole.
